# Understanding predation risk and individual variation in risk avoidance for threatened boreal caribou

**DOI:** 10.1002/ece3.3563

**Published:** 2017-10-25

**Authors:** Matthew A. Mumma, Michael P. Gillingham, Chris J. Johnson, Katherine L. Parker

**Affiliations:** ^1^ Ecosystem Science and Management University of Northern British Columbia Prince George BC Canada

**Keywords:** anthropogenic linear features, *Canis lupus*, learning, predation risk, *Rangifer tarandus*, resource selection function

## Abstract

Predation risk is a driver of species’ distributions. Animals can increase risk avoidance in response to fluctuations in predation risk, but questions remain regarding individual variability and the capacity to respond to changes in spatial risk across human‐altered landscapes. In northeast British Columbia, Canada, boreal caribou populations declined as roads and seismic lines have increased, which are theorized to increase gray wolf predation. Our goal was to model risk and to evaluate individual variability and the development of risk perception by examining individual risk avoidance in response to reproductive status and age. We used locations from collared caribou and wolves to identify landscape features associated with the risk of a potential wolf‐caribou encounter and risk of being killed given an encounter. We built resource selection functions to estimate individual responses to risk. We used general linear regressions to evaluate individual risk and linear feature avoidance as a function of age and reproductive status (calf or no calf). Linear features increased the risk of encounter. Older caribou and caribou with calves demonstrated stronger avoidance of the risk of encounter and roads, but weaker avoidance in late summer to the risk of being killed relative to younger and calf‐less individuals. Mechanisms explaining the inverse relationships between the risk of encounter and risk of being killed are uncertain, but it is conceivable that caribou learn to avoid the risk of encounter and roads. Responses by females with vulnerable calves to the risk of encounter and risk of being killed might be explained by a trade‐off between these two risk types and a prioritization on the risk of encounter. Despite the capacity to alter their responses to risk, the global decline in *Rangifer* populations (caribou and wild reindeer) suggests these behaviors are insufficient to mitigate the impacts of anthropogenic disturbances.

## 1 INTRODUCTION

Individual behaviors contribute to the competency of an individual to confront stressors (Brown, 2012). Beneficial traits should be more frequently passed to future generations, but environmental heterogeneity and stochasticity favor behavioral plasticity and the ability to alter behaviors in response to individual experiences (Brown, Ferrari, Elvidge, Ramnarine, & Chivers, 2013; Lima & Dill, 1990). Together these processes determine the ability of an individual to navigate multiple threats, while fulfilling nutritional needs (Hebblewhite & Merrill, 2009; Sih, 1992), which ultimately has direct implications for individual fitness (Creel, Winnie, Christianson, & Liley, 2008; LaMann & Martin, 2016).

Among threats, predation is understood to be an important influence on the behavior and distribution of many species (Lima & Dill, 1990). The threat of direct predation can decrease foraging efficiency by causing increased vigilance (Creel et al., 2008), but also can limit forage availability via the avoidance of risky habitats (Creel, Winnie, Maxwell, Hamlin, & Creel, 2005; Festa‐Bianchet 1988). These indirect effects of predation are hypothesized to increase individual stress under the “landscape of fear” hypothesis, and collectively, have the potential to decrease survival and reproduction (Laundré, Hernández, & Altendorf, 2001).

The mechanisms of predation risk are complex and often simplified for the purposes of study. Risk is frequently characterized as the probability of encounter (Eisenberg, Hibbs, & Ripple, 2015; Nicholson, Milleret, Månsson, & Sand, 2014), but for many species, risk is comprised of both the probability of encounter and the probability of being killed given an encounter (Fig. S1; Hebblewhite, Merrill, & McDonald, 2005; Heithaus & Dill, 2006). The probability of encounter is a function of the abundance and distribution of predators, along with local landscape features, which influence predator movement and predator detection of prey or vice versa (Lima & Dill, 1990). Thus, individual prey can experience dissimilar levels of risk as a result of their distribution and behavior (De Vos, O'Riain, Meyer, Kotze, & Kock, 2015; Hebblewhite & Merrill, 2009; Heithaus & Dill, 2006). An individual's behavior also can limit the probability of being killed given an encounter through the selection of habitats that provide ample escape terrain (Heithaus & Dill, 2006). Health and condition are additional determinants of whether or not an individual survives an encounter (Husseman et al., 2003).

Despite extensive research on the individual‐ and population‐level implications of predation, questions remain with regards individual variation in risk avoidance and the development of risk perception. Both evolution and learning contribute to predator recognition (Brown et al., 2013; Maloney & McLean, 1995), but deciphering the contributions of these mechanisms to spatial risk avoidance is challenging in natural systems. Prey living in the absence of historical predators can demonstrate prey naivety, but also rapid behavioral changes following predator reintroductions (Berger, 2007; Berger, Swenson, & Persson, 2001). The rapid nature of this response is consistent with the initiation of innate antipredator behaviors (Lima & Dill, 1990), but may also be the result of learning. Consistent with learning, older female cheetahs selected for areas with lower lion densities in comparison with younger individuals (Durant, 2000) suggesting that experience may allow older individuals to develop stronger responses to risk. Understanding age‐specific changes in risk avoidance, however, is further complicated by changes in the state of an individual (i.e., health status, Husseman et al., 2003; reproductive status, Cuiti, Bongi, Vassale, & Apollonio, 2006) and its environment. Thus, behavioral plasticity is essential for maximizing fitness across individual states and heterogeneous or changing landscapes (De Vos et al., 2015; Foam, Harvey, Mirza, & Brown, 2005; Ghalambor & Martin, 2002).

Our objectives were to determine landscape attributes that influence predation risk, examine individual variation in risk avoidance, and explore the development of risk perception for a species residing in a highly altered landscape. *Rangifer* populations (caribou and wild reindeer) are threatened globally from climate change and anthropogenic landscape alterations (Vors & Boyce, 2009), such as those resulting from logging, mining, and fossil fuel extraction (Cameron, Smith, White, & Griffith, 2005; Sorenson et al. 2008). In British Columbia (BC) and Alberta, Canada, these disturbances have decreased habitat quality and functional habitat quantity (Johnson, Ehlers, & Seip, 2015; Polfus, Hebblewhite, & Heinemeyer, 2011), but are also theorized to interact with predators (Latham, Latham, Boyce, & Boutin, 2011) and other prey species (apparent competition, Peters, Hebblewhite, DeCesare, Cagnacci, & Musiani, 2013) to increase predation on threatened boreal caribou (*Rangifer tarandus caribou*). Anthropogenic linear features, such as roads and seismic lines (cleared 3–10 m wide linear features resulting from natural gas exploration and ranging from 1 to 100 km in length), have been shown to increase gray wolf (*Canis lupus*) movement rates (Dickie, Serrouya, McNay, & Boutin, 2017), thereby increasing wolf search efficiency and wolf‐caribou encounters (Whittington et al., 2011). The influence of anthropogenic linear features on the probability of being killed remains unstudied and little is known regarding how prey species develop an appropriate response to novel landscape features that contribute to risk.

To explore predation risk and individual variability in risk avoidance, we first modeled spatial risk and then examined individual responses to risk by boreal caribou in northeast BC, Canada, as a function of age and reproductive status (calf or no calf). Risk in this system might be further complicated by the recent increase in anthropogenic disturbances (<25 years) for which caribou lack an evolutionary history; therefore, we also separately assessed individual responses to roads and seismic lines. Given the affinity of wolves for anthropogenic linear features (Dickie et al., 2017; Whittington et al., 2011), we hypothesized (1) that the risk of encounter would be increased near roads and seismic lines. We also predicted that (2) risk avoidance would increase for older females, consistent with the greater experience of older individuals in navigating through high‐ and low‐risk areas, and for females with calves, as they seek to limit predation risk for vulnerable offspring. Further, we anticipated (3) that the greater experience of older individuals would lead to stronger avoidance of roads and seismic lines in comparison with younger individuals. In this study, we demonstrate high individual variability in risk avoidance and the capacity of individuals to adjust their responses to risk in a landscape extensively altered by anthropogenic disturbances.

## 2 MATERIALS AND METHODS

### 2.1 Study site and animal capture

**Figure 1:**
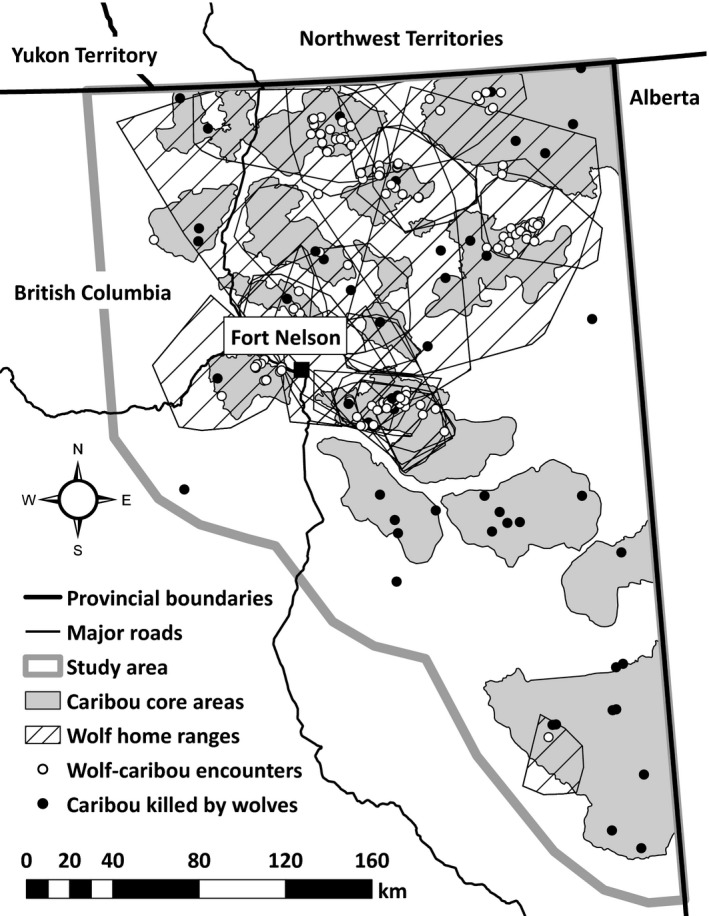
Map of study area in northeast British Columbia detailing boreal caribou core areas, wolf home ranges (100% minimum convex polygons), potential wolf‐caribou encounters, and caribou mortality sites attributed to wolves from December 2012 until spring 2016

The northeastern BC landscape contains deciduous and mixed‐wood uplands, vast peatland complexes, and riparian areas (Delong, Annas, & Stewart, 1991) with elevations ranging from 214 to 1,084 m. The area has a northern continental climate that consists of cold winters and short summers (Environment Canada 2016). Anthropogenic disturbance is widespread, primarily due to natural gas development, but also as a result of logging. Roads and seismic lines are particularly evident (Figs S2 and S3) and overlap caribou core areas. Core areas (Figure 1) were previously delineated using aerial surveys and locations of collared individuals and correspond to primary‐use areas for individual herds (Ministry of Environment 2016). The density of roads and seismic lines within core areas ranges from 420–1,357 m/km^2^ to 43–6,810 m/km^2^, respectively.

During the winters between December 2012 and April 2015, 223 female caribou and 32 wolves were captured and affixed with radio (115 caribou) and global positioning system (GPS; 108 caribou and 32 wolves) collars using aerial net gunning in accordance with approved BC government guidelines (Resources Inventory Committee, 1998) and institutional animal care protocols (BC Wildlife Act Permits FJ12‐76949 and FJ12‐80090). At time of capture, caribou were assigned ages based on tooth wear, and samples were collected to determine pregnancy status using progesterone tests (Ropstad et al., 1999). Collared animals were monitored regularly, and when applicable and feasible, mortality locations were investigated to determine the cause of death from evidence of predation (i.e., bite marks, predator scats, disturbed vegetation indicating a struggle), disease, starvation, or human causes (i.e., harvest, vehicle collision). Late winter surveys were also conducted each year to determine which females had successfully raised a calf. See supporting information (collar models and monitoring) for additional details.

### 2.2 Modeling predation risk

Our first step in modeling risk was to identify potential wolf‐caribou encounters. We used individual locations for 97 GPS‐collared caribou with the remaining individuals being excluded due to collar malfunction, individuals leaving the study area, or death within 1 month of collaring. We used locations for 28 collared wolves and excluded the four remaining collars because of a limited number of fixes resulting from collar failures. We eliminated caribou and wolf locations <48 hr following capture and screened data for spurious locations. We identified potential wolf‐caribou encounters using program R (R Core Team 2015) by determining caribou locations where a wolf was within 1,971 m in a 24‐hr moving window, similar to other studies (Bastille‐Rousseau et al., 2016; Creel et al., 2005; Gude, Garrott, Borkowski, & King, 2006; Muhly et al., 2010; Whittington et al., 2011). Our criterion was based on the mean of 24‐hr wolf movement distances. We included multiple encounters per individual when applicable, but eliminated all but the first potential encounter if multiple encounters were detected for the same individual within a 24‐hr window. Because our criterion could not assure that a wolf‐caribou encounter actually occurred, we considered those caribou locations as potential wolf‐caribou encounters, representing locations where a wolf was in close proximity to a caribou, but the caribou evaded death directly (actual encounter and escape) or indirectly by avoiding detection.

We compared potential wolf‐caribou encounters to all caribou locations to determine the probability of an encounter. We then modeled the probability of being killed by contrasting potential wolf‐caribou encounters with collared caribou mortality sites attributed to wolves, which represented fatal encounters (Fig. S1). Anticipating seasonal differences, we divided our dataset between snow‐free (16 May–31 October) and snow periods (1 November–15 May) and built competing models for each probability by season using a suite of covariates that were generated in ArcGIS (ESRI 2015) and theorized to influence risk. We tested all covariates for collinearity via QR decomposition (package caret, Kuhn et al., 2013). We used logistic regression to estimate model coefficients and then used Akaike's information criteria for small sample sizes (AICc) to determine the most parsimonious (lowest AICc, Tables S2–S5) models (Burnham & Anderson, 2002; package MuMIn, Bartón, 2015). We used the ROC test to assess model fit (Mason & Graham, 2002; package pROC, Robin et al., 2011). All statistical analyses were completed in program R (R Core Team 2015).

Models for the probability of encountering a wolf included both natural and anthropogenic landscape covariates. We reclassified 30 landscape classes from a Ducks Unlimited Vegetation Layer (30 × 30‐m resolution; Ducks Unlimited, Inc. 2010) into eight vegetation classes (Wilson & DeMars, 2015) using ArcGIS (ESRI 2015). These classes were included as categorical covariates and consisted of conifer swamp, hardwood swamp, nutrient‐poor fen, nutrient‐rich fen, treed bog, upland conifer, upland deciduous, and a reference category (other), which included rocky uplands, anthropogenic, burns, several aquatic classes, and areas obscured by clouds (see Wilson & DeMars, 2015 for vegetation‐class descriptions). We also determined the elevation (m), slope (degrees), and distance (m) to water and calculated the proportions of hardwood swamp, treed bog, and nutrient‐poor and rich fens combined within a 100‐m radius around each potential wolf‐caribou encounter location and each caribou location. We specifically selected these four vegetation classes because of their known preference by moose (*Alces alces*; hardwood swamp, Mumma & Gillingham, 2016) or caribou (treed bog, nutrient‐poor fen, and nutrient‐rich fen, Wilson & DeMars, 2015) in the boreal ecosystem during certain seasons. Previous studies demonstrate that wolves select for areas with greater landscape complexity and fragmentation (Houle, Fortin, Dussault, Courtois, & Ouellet, 2010; Milakovic et al., 2011). We, therefore, included vegetation‐class diversity (Shannon Index, Shannon, 1948), density of vegetation‐class edges (m/km^2^), and terrain roughness at a 100‐m radius. Terrain roughness was calculated as the standard deviation of all slopes (Grohmann, Smith, & Ricconini, 2011) within a 100‐m radius. Because the distribution of terrain roughness was high, right‐skewed, we placed values into 20 bins separated by 19 equally spaced quantiles (5%–95%). We calculated the mean value of locations within each bin and then assigned the mean terrain roughness value of each bin to the corresponding locations. Covariates of anthropogenic disturbance included the densities (m/km^2^) of roads and seismic lines within a 100‐m radius and distances (m) to roads and seismic lines.

Because caribou mortality sites attributed to wolves likely involved a chase, we only included covariates at a 100‐m radius of each caribou mortality site and potential wolf‐caribou encounter, but also evaluated covariates at a 500‐m radius to determine if our models were robust to changes across scales. Covariates included the proportions of hardwood swamp, treed bog, nutrient‐poor and rich fens combined, vegetation‐class diversity (Shannon Index, Shannon, 1948), density (m/km^2^) of vegetation‐class edges, terrain roughness (Grohmann et al., 2011), density (m/km^2^) of roads, and density (m/km^2^) of seismic lines. All landscape covariates were generated using ArcGIS (ESRI 2015).

### 2.3 Evaluating individual variation in risk avoidance

To evaluate individual variation in risk avoidance by reproductive status and age, we first used mixed‐effects logistic regression (i.e., Johnson, Nielsen, Merrill, McDonald, & Boyce, 2006; package lme4, Bates, Maechler, Bolker, & Walker, 2015) to estimate resource selection functions in a use‐available design (RSF, Boyce, Vernier, Nielson, & Schmiegelow, 2002). A RSF (*w*) is proportional to the relative probability of selection, in our case, by relating model covariates (**x **= *x*_1_, *x*_2_, …, *x*_n_) to the covariate values at individual locations. We limited our

(1) $$\text{RSF}=w\left(x\right)=\exp\left(\beta_{1}x_{1}+\beta_{2}x_{2}+\cdots +\beta_{n}x_{n}\right)$$

analyses to prime‐aged (2–10 years, Skogland, 1985) individuals (*n* = 84) to ensure representation for each age and because of our increased uncertainty in estimating the age of older individuals. We partitioned our data into four seasons based on caribou life history (calving 16 May–15 July, late summer 16 July–31 October, early winter 1 November–31 January and late winter 1 February–15 May). We determined resource availability by buffering each used location by the 90% centile of movement distances for each individual by season and then selecting five random locations from within that buffer. Recognizing that species balance risk with other factors, such as forage quality or quantity, we first built RSFs including the eight previously delineated vegetation classes as categorical covariates using deviation coding, with other as a reference category, along with a random intercept for each individual by year. Next, we used the most parsimonious risk model for the snow‐free period (see Section 2.2) to predict the relative probabilities of the risk of encounter and the risk of being killed for each used and available location during calving and late summer. The most parsimonious risk model for the snow period (see Section 2.2) was used to predict the risk of encounter and risk of being killed during early and late winter. To the vegetation‐class base model of each season, we added risk covariates (predicted probability of encounter and predicted probability of being killed) as random slopes for each individual by year. This modeling approach allowed us to estimate individual responses to risk for each individual for each year an individual was active in the study by estimating individual coefficients (β) to the risk of encounter and risk of being killed. We used AICc to establish that vegetation‐class base models were improved via the inclusion of risk covariates and verified model fit through k‐fold cross‐validation using 10 repetitions of fivefold cross‐validation with 10 bins of equal size (Boyce et al., 2002).

We then built two general linear regression models for each season using the βs estimated for the risk of encounter and the risk of being killed for each individual by year as dependent variables. We modeled age as a continuous covariate and reproductive status (calf or no calf) as a categorical covariate in each model. To capture the change in age for individuals that survived their first year postcollaring and whose collars remained active, we added an additional year to the estimated age at capture for each additional year they remained active (sometimes 2 years and rarely three). Although we knew pregnancy status for the first year postcapture, pregnancy status in the second and third years was unknown. To address this limitation, we assigned females that tested negative for pregnancy as without calves (no calf group) and grouped pregnant females and females with an unknown pregnancy status within a calf group for the calving season. We assumed that most of the untested females would have been pregnant, as pregnancy rates of tested females were high (>85%). Because caribou calf vulnerability is highest during the calving season (Gustine, Parker, Lay, Gillingham, & Heard, 2006), we inferred that females observed without calves during late winter surveys were most likely to have lost their calves to predation or other causes prior to the late summer season. We, therefore, assigned reproductive status (calf or no calf) during late summer and early and late winter based on the results (calf or no calf) of the late winter surveys. Although we recognized that some females were likely assigned to the wrong group (calf or no calf), we thought our approach was the best means of incorporating some of the individual variability in risk avoidance associated with reproductive status. Although health and condition likely impacted species responses to risk, we lacked sufficient information to incorporate this additional source of variability. We interpreted significance for age and reproductive status using *p*‐values (≤.1). When only one covariate was significant, we excluded the nonsignificant covariate and ran univariate models including the remaining significant covariate.

### 2.4 Evaluating individual variation in responses to roads and seismic

We used a similar approach to evaluate age‐specific avoidance of roads and seismic lines. We first built RSFs (Boyce et al., 2002) for each season including eight vegetation classes and a random intercept for individual by year. We then built two additional models for each season by adding, to the vegetation‐class base models, the densities (m/km^2^) of roads and seismic lines at a 100‐m radius and the distances (m) to roads and seismic lines. We established model improvement over the vegetation‐class base model using AICc and verified model fit via k‐fold cross‐validation using 10 repetitions of fivefold cross‐validation with 10 bins of equal size (Boyce et al., 2002).

We then built four general linear regression models for each season using the βs estimated for the densities (m/km^2^) of and distances (m) to roads and seismic lines for each individual by year as dependent variables. Age was included as a continuous covariate and reproductive status (calf or no calf) as a categorical covariate in each model. We interpreted significance using *p*‐values (≤.1) and excluded the nonsignificant covariate and reran models with the remaining significant covariate, when applicable. All statistical analyses were completed in program R (R Core Team 2015).

## 3 RESULTS

### 3.1 Predation risk models

We used 96,594 caribou and 20,735 wolf locations to identify the locations of 57 and 55 potential wolf‐caribou encounters (Figure 1) during the snow‐free and snow periods, respectively. Vegetation‐class diversity and density of vegetation‐class edges were collinear and therefore not included together in any candidate models. The best models for predicting the probability of encounter during snow‐free and snow periods shared many of the same covariates (Tables S1 and S2). A negative relationship with elevation and positive relationships to the proportions of hardwood swamp and treed bog, along with positive relationships to the densities of roads and seismic lines, were included in the snow‐free model (Table 1). In the snow model, elevation remained negative and positive relationships remained for the proportions of hardwood swamp and treed bog, but replaced the density of roads and seismic lines with the distance to roads and seismic lines (Table 1).

**Table 1:** The coefficients (β) and standard errors (*SE*) for each covariate (standardized between 0 and 1) in the most parsimonious models of the probability of encountering (Prob. encounter) a wolf and the probability of being killed (Prob. being killed) given an encounter in snow‐free and snow seasons for boreal caribou in northeast British Columbia. Proportion (Prop.) and density (m/km^2^) metrics calculated within a 100‐m radius. Edge density is the density of vegetation‐class edges and terrain roughness refers to the binned standard deviation of slopes within a 100‐m radius. Distance (m) = dist

	Snow‐free		Snow	
	β	*SE*	β	*SE*
Prob. encounter				
Intercept	−6.894	0.421	−5.677	0.493
Prop. hardwood swamp	0.980	0.941	1.352	0.949
Prop. bog	1.162	0.412	0.792	0.426
Elevation	−5.938	2.690	−2.705	0.828
Road density	1.791	1.307		
Seismic density	1.998	0.868		
Dist. to roads			−5.181	1.805
Dist. to seismic			−3.369	1.467
Prob. being killed				
Intercept	−0.907	0.477	1.341	1.150
Prop. swamp	3.970	1.864	0.528	1.582
Prop. bog			−2.633	1.211
Prop. fen			−2.056	1.423
Edge density	−3.208	1.381		
Terrain roughness	2.414	1.718		

The snow‐free model included the proportions of hardwood swamps and treed bogs and elevation, along with the density of roads and seismic lines (Table 1). The most parsimonious model during the snow period also contained the proportions of hardwood swamps and treed bogs and elevation, but replaced the density of roads and seismic lines with the distance to roads and seismic lines (Table 1). The ROC values (snow‐free ROC = 0.711, 95% CI = 0.654–0.767 and snow ROC = 0.683, 95% CI = 0.613–0.752) indicated these models were reasonably predictive.

We compared potential wolf‐caribou encounters (57 snow‐free and 55 in the snow period) with caribou mortality sites attributed to wolves (20 snow‐free, 35 snow) to model the probability of being killed (Figure 1). The most parsimonious models were consistent across scales (100‐m and 500‐m radii), but differed between snow‐free and snow periods (Tables S3–S6). In the snow‐free period, a higher proportion of hardwood swamps increased the probability of being killed as did locations with greater terrain roughness, whereas areas with greater densities of vegetation‐class edges resulted in a lower probability of being killed (Table 1). During the snow period, higher proportions of hardwood swamps increased the probability of being killed, whereas higher proportions of treed bogs and fens decreased the probability of being killed (Table 1). The ROC value for our snow‐free model was 0.738 (95% CI = 0.632–0.844) and for our snow model was 0.731 (95% CI = 0.605–0.856).

### 3.2 Individual variation in risk avoidance

**Figure 2:**
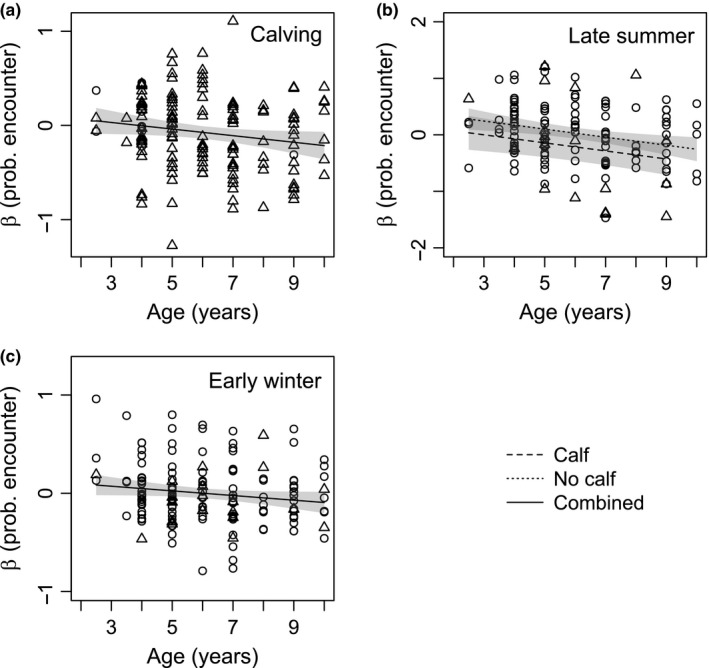
Individual coefficients (β) for the predicted probability of encountering (prob. encounter) a wolf (from resource selection functions) in calving (a), late summer (b), and early winter (c) seasons as a function of age and reproductive status (calf or no calf) for boreal caribou in northeast British Columbia. A single combined curve is shown in calving and early winter, because reproductive status was not significant (*p*‐value <.1). Triangles (Δ) indicate βs for caribou with calves, and circles (○) indicate caribou without calves

**Figure 3:**
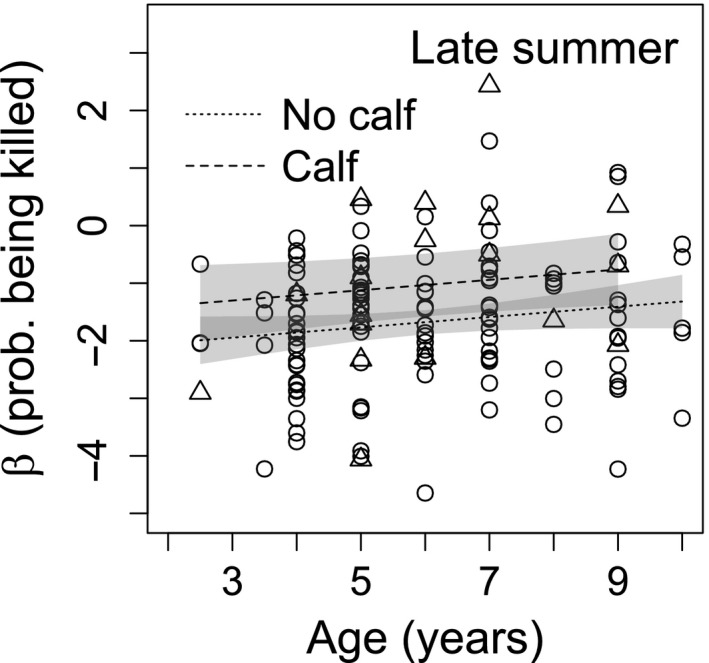
Individual coefficients (β) for the predicted probability of being killed (prob. being killed) given a wolf encounter (from a resource selection function) in late summer as a function of age and reproductive status (calf or no calf) for boreal caribou in northeast British Columbia. Triangles (Δ) indicate βs for caribou with calves, and circles (○) indicate caribou without calves

Resource selection functions were improved via the inclusion of risk covariates in all seasons (Table S7). Across seasons, caribou consistently selected for treed bogs and conifer swamps and avoided uplands (deciduous and conifer; Table 2). During calving, caribou also selected for hardwood swamps and fens (nutrient‐poor and rich), but avoided hardwood swamps in late summer, while continuing to select nutrient‐poor and rich fens (Table 2). During early winter, hardwood swamps and nutrient‐rich fens were selected and nutrient‐poor fens were avoided (Table 2). In late winter, hardwood swamps and nutrient‐poor fens were selected and nutrient‐rich fens were avoided (Table 2). General linear regressions revealed stronger avoidance to the risk of encounter for older individuals during calving, late summer, and early winter (Table S8; Figure 2), but weaker avoidance to the risk of being killed in late summer (Table S9; Figure 3). In late summer, calf presence increased avoidance of the risk of encounter (Table S8; Figure 2b), but decreased avoidance of the risk of being killed (Table S9; Figure 3).

**Table 2:** The coefficients (β) and standard errors (*SE*) of fixed effects and the variance (σ) of random effects in resource selection functions including risk covariates (predicted probability of encountering a wolf and predicted probability of being killed given an encounter) during calving, late summer, early winter, and late winter seasons for boreal caribou in northeast British Columbia. Nutrient‐poor fen = poor fen, nutrient‐rich fen = rich fen, probability of encountering a wolf = Prob. encounter, probability of being killed given an encounter = Prob. being killed

Fixed effects	Calving		Late summer	
	β	*SE*	β	*SE*
Conifer swamp	0.257	0.040	0.280	0.031
Hardwood swamp	0.164	0.042	−0.136	0.034
Poor fen	0.661	0.025	0.512	0.018
Rich fen	0.384	0.035	0.087	0.028
Treed bog	0.716	0.025	0.673	0.018
Upland conifer	−0.584	0.060	−0.357	0.042
Upland deciduous	−1.345	0.097	−1.374	0.064

Random effects	σ		σ	
Prob. encounter	0.389		0.574	
Prob. being killed	4.880		4.657	
Individual	0.120		0.108	

Fixed effects	Early winter		Late winter	
	β	*SE*	β	*SE*
Conifer swamp	0.030	0.038	0.161	0.031
Hardwood swamp	0.132	0.037	−0.125	0.038
Poor fen	−0.068	0.027	0.268	0.023
Rich fen	0.153	0.032	−0.213	0.032
Treed bog	0.253	0.031	0.412	0.027
Upland conifer	−0.217	0.053	−0.036	0.043
Upland deciduous	−1.187	0.079	−0.866	0.064

Random effects	σ		σ	
Prob. encounter	0.146		0.159	
Prob. being killed	5.079		4.057	
Individual	0.013		0.061	

### 3.3 Individual variation in responses to roads and seismic

**Figure 4:**
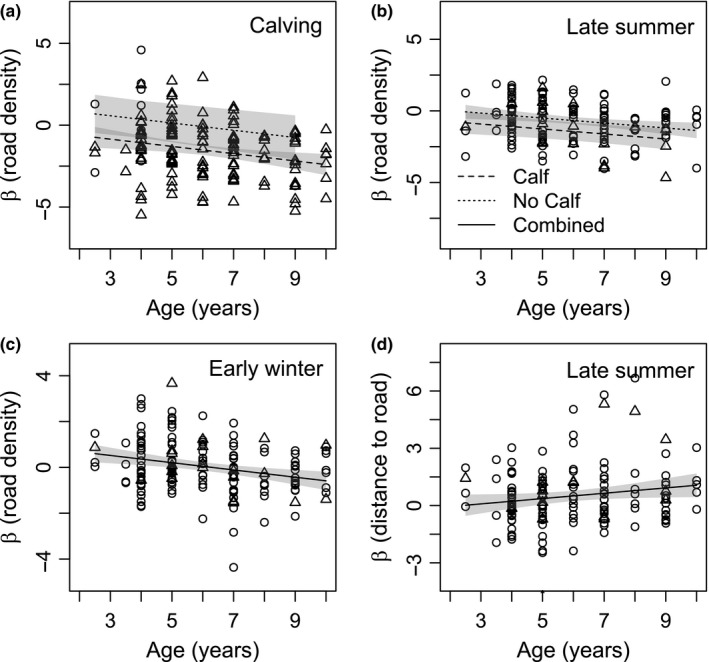
Individual coefficients (β) for the density (m/km^2^) of roads (from resource selection functions) in calving (a), late summer (b), and early winter (c) and for the distance (m) to roads (from a resource selection function) in late summer (d) as a function of age and reproductive status (calf or no calf) for boreal caribou in northeast British Columbia. A single combined curve is shown in early and late winter, because reproductive status was not significant (*p*‐value <.1). Triangles (Δ) indicate βs for caribou with calves, and circles (○) indicate caribou without calves

The inclusion of road and seismic line covariates improved RSFs across seasons (Table S10), but less than the inclusion of risk covariates with the exception of the calving season (Tables S7 and S10). In calving and late summer, responses to vegetation classes were consistent between RSFs containing risk covariates and RSFs containing road and seismic line covariates (Tables 2, S11 and S12). There were differences, however, in some of the responses to swamps and fens in early and late winter (Tables 2, S11, and S12). General linear regression models revealed stronger avoidance to the density of roads by older caribou during calving, late summer, and early winter (Table S13, Figure 4a–c). Older caribou also demonstrated increased selection for areas further from roads in comparison with younger caribou in late summer (Table S14, Figure 4d). During calving, calf presence increased the avoidance of areas with high densities of roads and seismic lines and areas near roads (Tables S13–S15, Figure 4a) and increased the avoidance of areas with high densities of roads during late summer (Table S13, Figure 4b). No relationships were detected in any season for the distance to seismic lines as a function of age or reproductive status (Table S16).

## 4 DISCUSSION

Our findings suggest that roads and seismic lines increase the risk of a wolf‐caribou encounter and demonstrate individual variation in risk avoidance partially dependent upon age and reproductive status. Consistent with our first hypothesis, being near or in areas with higher densities of roads and seismic lines increased the probability of encountering a wolf (Table 1). We did not find any relationship between anthropogenic linear features and the probability of being killed given an encounter (Table 1). Stronger avoidance by older individuals and individuals with calves to the risk of encounter (Figure 2) and to areas with roads (Figure 4) aligned with our second and third hypotheses. These findings suggest that older individuals and individuals with calves are more risk‐averse than younger individuals or individuals without calves. The weaker responses in late summer, however, by older individuals and individuals with calves to areas with a higher probability of being killed (Figure 3) would seem to support an opposing conclusion.

The development of risk perception is one potential explanation for the relationship between age and an individual's response to the risk of encounter and roads. During calving, late summer, and early winter, older caribou demonstrated stronger risk avoidance to areas with a higher risk of encountering a wolf and to areas with higher road densities (Figures 2, 4a–c). Older caribou also more strongly avoided areas near roads in late summer (Figure 4d). These trends might result from caribou learning to minimize the risk of encounter with age. Potential mechanisms by which learning could occur include the utilization of spatial and attribute memory (Fagan et al., 2013). Through experience, caribou might develop a spatial map of their environment and avoid areas frequented by wolves (spatial memory). Alternatively, caribou might learn to associate landscape features, such as roads, with wolves and subsequently avoid these features when encountered (attributed memory).

Other explanations linking risk avoidance and age are also plausible. Through time, caribou with weaker risk avoidance might be selected out (killed by wolves) of the population leaving only individuals with strong, risk‐averse responses. Under this scenario, we would expect that higher individual variation in risk avoidance would be present for younger age classes and that variation would decrease with age, leaving mainly individuals with higher risk avoidance. The high and relatively consistent variation across age classes does not support this explanation (Figure 2). Differences in condition or nutritional needs might also generate differences in risk avoidance by altering an individual's optimal trade‐off between forage and predation risk. As we limited our RSF analyses to prime‐aged individuals (2–10 years), a linear relationship between age and condition is unlikely (Skogland, 1985), although differences in individual condition, regardless of age, likely explains some of the unexplained variability in risk avoidance (Figure 2). Nutritional needs are increased for lactating females with calves (Gerhart, Cameron, & Russell, 1996) and 2‐year‐old females had lower pregnancy rates in our study (Figure 2), but a linear increasing relationship across ages with reproductive status was not present. Further, we accounted for reproductive status (calf or no calf) in our models.

Lactating females must balance high nutritional demands with high calf vulnerability. Cuiti et al. (2006) demonstrated altered space‐use for fallow deer (*Dama dama*) resulting from the presence of offspring. We anticipated that reproductive status would be most influential during the calving season when calves are most vulnerable (Gustine et al., 2006), but did not find a relationship between risk avoidance and reproductive status during calving (Tables S8 and S9). Our imperfect knowledge of calf presence (see Section 2.3) might account for the absent relationship, although we did find that females with calves more strongly avoided areas with higher densities of roads and seismic lines during the calving season (Figure 4a, Table S15). We also observed that females with calves avoided areas with a higher risk of encounter and areas containing higher road densities in late summer (Figures 2b and 4b).

We remain uncertain with regard to the inverse relationships observed in late summer between the risk of encounter and the risk of being killed in response to age and reproductive status (Figures 2b and 3). A trade‐off between the risk of encounter and risk of being killed is a potential explanation. We did not detect correlations between the probabilities of encounter and probabilities of being killed, but did detect a positive relationship between terrain roughness and the risk of being killed in the snow‐free season (Table 1). In contrast, other studies observed increased caribou calf survival in areas with greater terrain roughness and theorized that these areas decreased predator encounters (Bergerud, Butler, & Miller, 1984; Pinard, Dussault, Ouellet, Fortin, & Courtois, 2012). Even though the risk of encounter was better explained by other covariates, a weak relationship between terrain roughness and the risk of encounter (Table S1) might necessitate weaker responses to areas with a high risk of being killed as a result of a stronger avoidance to the risk of encounter.

Indeed, a stronger avoidance to the risk of encounter may have a greater benefit for caribou survival than a stronger avoidance to areas with a high risk of being killed. The degree of spatial separation between a prey and predator species is in part a function of the ability of the prey to escape a predator encounter (Wirsing, Cameron, & Heithaus, 2010). Boreal caribou space‐use is characterized by a spacing‐away strategy by which individuals or small groups of caribou disperse widely across their landscape (Bergerud & Page, 1987). This behavior is hypothesized to reduce predator encounters (DeMars, 2015); thus, one could deduce that boreal caribou have a limited capacity to evade predators once encountered and that reducing the risk of encounter is likely more important for caribou survival than reducing the risk of being killed per se. Females with calves likely prioritize avoidance of areas with a high risk of encounter (Figure 2b) over the risk of being killed (Figure 3) given the high vulnerability of calves and their lower likelihood of evading death given an encounter. There is not, however, a clear explanation for why older prime‐aged caribou during late summer also seems to be prioritizing the risk of encounter (Figures 2b and 3) in comparison with younger prime‐aged individuals that are expected to have a comparable capacity to escape a wolf‐caribou encounter.

The probability of an encounter within a specific habitat should be proportional to the time spent by the prey and predator species within that habitat (Hebblewhite et al., 2005). Therefore, it is not surprising that encounters increased near roads and seismic lines (Table 1), given the propensity of wolves to select for anthropogenic linear features (Dickie et al., 2017). Likewise, increased encounters at lower elevations (Whittington, St, Clair, & Mercer, 2005) and in areas with higher proportions of hardwood swamps (Table 1), which are selected by an alternative prey species—moose (Mumma & Gillingham, 2016), are also consistent with our understanding of wolf use and selection. The positive relationship between the risk of encounter and the proportion of treed bogs was unexpected (Table 1). In northeast BC, caribou spend most of their time in treed bogs (Wilson & DeMars, 2015), and peatlands, such as treed bogs, are thought to provide caribou with a refugium from predators (Latham, Latham, McCutchen, & Boutin, 2011).

There was additional uncertainty in the mechanisms explaining our most parsimonious models for the probability of being killed. Given that our encounters were not true encounters, but instead represented the potential for a caribou to encounter a wolf, it is conceivable that the potential for a true encounter (actual interaction between a caribou and wolf) and a caribou mortality event was increased when an encounter occurred in an area frequently used by wolves. For example, the probability that a wolf actually detects a caribou following a potential wolf‐caribou encounter might be increased if that caribou is occupying a habitat with a high probability of wolf use (i.e., hardwood swamp, Table 1) in comparison with a habitat with a low probability of wolf use (i.e., treed bog or fens, Table 1). Thus, our modeling approach for the probability of being killed may be much more contingent upon the detection process than the process by which a caribou escapes or succumbs following a true encounter. This highlights the numerous mechanisms that underlie risk, and although the two‐step process of risk (risk of encounter and risk of being killed given an encounter, Hebblewhite et al., 2005) is a reasonable compartmentalization, a further division into the probability of a potential encounter, probability of a detection given a potential encounter, and the probability of being killed given a detection may better approximate the risk process.

The decreased probability of being killed in areas with higher densities of vegetation‐class edges (edge density, Table 1) may also be reflective of the detection step in the risk process. Greater habitat complexity resulting from higher edge density may limit wolf visibility and movement, although the impediment to movement could also aid in the ability of caribou to escape a true encounter. Intuitively, the increased probability of being killed as terrain roughness increases (Table 1) also seems more likely to be related to the escape process. Further, contrasting space‐use patterns between boreal caribou (open, relatively flat peatlands, Wilson & DeMars, 2015) and wolves (more complex landscapes, Houle et al., 2010; Milakovic et al., 2011) suggests potential differences between the species in their ability to navigate landscapes with higher terrain roughness.

## 5 CONCLUSIONS

Controlled experiments have provided insights into how animals learn to recognize and respond to predators (Ferrari, 2014; Griffin & Evans, 2003), and studies in natural settings have demonstrated the ability of individuals to respond to temporal and spatial changes in predation risk (Berger, 2007; De Vos et al., 2015). Our study first characterized spatial predation risk and then explored individual variability in risk avoidance as a function of age and reproductive status. We found that anthropogenic linear features increased the probability of a wolf‐caribou encounter, which was consistent with previous research (DeMars, 2015; Whittington et al., 2011). We also detected stronger responses for older caribou and caribou with calves to the risk of encounter in comparison with younger individuals and individuals without calves, but weaker responses in late summer to the risk of being killed by older individuals and individuals with calves. The mechanisms explaining the inverse relationships between the risk of encounter and risk of being killed in response to age are unknown, but it is conceivable that caribou might be capable of learning to avoid areas with a higher risk of encounter and higher road densities. The observed responses for females with calves to the risk of encounter and the risk of being killed are likely explained by females with vulnerable calves prioritizing the risk of encounter over the risk of being killed, particularly if a trade‐off exists between these two types of risk. The behavioral plasticity observed in this study demonstrates that caribou are capable of modifying behaviors in response to risk, including the increased risk associated with roads, but global declines in the abundance of caribou and reindeer (Vors & Boyce, 2009) indicate that caribou behavioral alterations are likely insufficient to compensate for the negative impacts of anthropogenic disturbances.

## DATA ACCESSIBILITY

Data are currently accessible through the BC Oil and Gas Research and Innovation Society (http://www.bcogris.ca/boreal-caribou/projects/active).

## CONFLICT OF INTEREST

None declared.

## AUTHOR CONTRIBUTIONS

MM conceived the ideas, conducted the analysis, and wrote the manuscript. MG, CJ, and KP provided funding, along with theoretical and analytical guidance and manuscript edits.
