# Supplementary material for: Understanding predation risk and individual variation in risk avoidance for threatened boreal caribou
Source: Ecol Evol. 2017 Oct 25;7(23):10266–77. doi: 10.1002/ece3.3563 (PMC5723594; doi:10.1002/ece3.3563)
Supplement: Supplementary file 1 [file ECE3-7-10266-s001.docx]

**Understanding predation risk and individual variation in risk avoidance for threatened boreal caribou**

Matthew A. Mumma^1*^, Michael P. Gillingham^1^, Chris J. Johnson^1^, and Katherine L. Parker^1^

**Supporting information**

COLLAR MODELS AND MONITORING

Lotek LMRT4 Very High Frequency (VHF) collars (Lotek Wireless Inc., Newmarket, Ontario) and several types of GPS collars were deployed on 115 and 108 female caribou, respectively. GPS collars included Lotek Lifecycle GlobalStar (Lotek Wireless Inc., Newmarket, Ontario), ATS Iridium GPS G2110E (Advanced Telemetry Systems Inc., Isanti, Minnesota), and Vectronic Vertex Survey GlobalStar (Vectronic Aerospace, Berlin, Germany). The rate at which individual collars transmitted caribou locations varied (4, 8, 11, 12, and 13 h) and changed seasonally for some collars. Wolf collars included Lotek Iridium TrackM (Lotek Wireless Inc., Newmarket, Ontario) and Vectronic Vertex Survey Iridium (Vectronic Aerospace, Berlin, Germany); transmission rates varied (0.25, 1, 2, 3, 4, 8, and 23 h) between collars and seasonally. Caribou collared with VHF collars required monthly monitoring flights to determine collar status, whereas caribou and wolves with GPS collars could be monitored remotely. Regardless of collar type, motionless collars were checked to determine individual status.

**Figures**

FIG. S1. Conceptual model for the probability of encounter and the probability of being killed.

FIG. S2. Map of public, forest service, and resource (logging or natural gas access) roads in northeast British Columbia.

FIG. S3. Map of seismic lines in northeast British Columbia. Seismic lines are created during the natural gas exploration process and consist of linear features 3–8 m in width that have been cleared of trees and shrubs. Gray polygons represent areas of dense (100–500 m apart) 3D seismic.

**Tables**

TABLE S1. Snow-free season candidate models for the probability of encountering a wolf (Prob. of encounter) for woodland caribou in northeast British Columbia evaluated using Akaike’s Information Criteria for small sample sizes (AICc). Interactions indicated by *. Conifer swamp = conifswamp, hardwood swamp = hardswamp, nutrient-poor fen = poorfen, nutrient-rich fen = richfen, treed bog = bog, upland decidious = updecid, upland conifer = upconif, proportion of hardwood swamp within 100 m = hardswamp100, proportion of treed bog within 100 m = bog100, proportion of nutrient-poor fen within 100 m = poorfen100, nutrient-rich fen within 100 m = richfen100, terrain roughness (standard deviation of slopes) within 100 m = tr100, vegetation class diversity within 100 m = vcd100, density (m/km^2^) of vegetation class edges within 100 m = edge100, density (m/km^2^) of roads within 100 m = road100, distance (m) to road = distroad, density (m/km^2^) of seismic lines within 100 m = seismic100, distance (m) to seismic line = distseismic, distance (m) to vegetation class edge = distedge, distance (m) to water = distwater, number of parameters = k

| Prob. encounter models – snow-free | k | Log-likelihood | AICc | Delta AICc |
| --- | --- | --- | --- | --- |
| Prob. encounter=hardswamp100+bog100+elevation+seismic100+road100 | 6 | -426.1 | 864.1 | 0.0 |
| Prob. encounter=hardswamp100+bog100+elevation+distseismic+distroad | 6 | -426.2 | 864.3 | 0.2 |
| Prob. encounter=hardswamp100+bog100+distwater+elevation+tr100+seismic100+distseismic | 8 | -425.4 | 866.7 | 2.6 |
| Prob. encounter=hardswamp100+bog100+distwater | 4 | -429.9 | 867.7 | 3.6 |
| Prob. encounter=hardswamp100+bog100+distseismic+seismic100 | 5 | -429.2 | 868.4 | 4.2 |
| Prob. encounter=hardswamp100+bog100 | 3 | -431.5 | 868.9 | 4.8 |
| Prob. encounter=hardswamp100+bog100+distwater+road100+distroad+seismic100+distseismic | 8 | -427.0 | 869.9 | 5.8 |
| Prob. encounter=hardswamp100+bog100+distwater+elevation+tr100+slope | 7 | -428.2 | 870.4 | 6.3 |
| Prob. encounter=hardswamp100+bog100+distroad+road100+distseismic+seismic100 | 7 | -428.3 | 870.5 | 6.4 |
| Prob. encounter=hardswamp100+bog100+vcd100 | 4 | -431.4 | 870.8 | 6.7 |
| Prob. encounter=hardswamp100+bog100+distwater+distedge+vcd100 | 6 | -429.7 | 871.4 | 7.3 |
| Prob. encounter=poorfen100+richfen100+bog100 | 4 | -431.8 | 871.7 | 7.5 |
| Prob. encounter=hardswamp100+bog100+distedge+edge100 | 5 | -431.1 | 872.3 | 8.1 |
| Prob. encounter=(poorfen100*seismic100)+(richfen100*seismic100)+(bog100*seismic100) | 8 | -429.5 | 874.9 | 10.8 |
| Prob. encounter=slope+tr100+elevation | 4 | -433.5 | 874.9 | 10.8 |
| Prob. encounter=road100+distroad+distseismic+seismic100 | 5 | -434.6 | 879.1 | 15.0 |
| Prob. encounter=conifswamp+hardswamp+poorfen+richfen+bog+updecid+upconif | 8 | -433.2 | 882.3 | 18.2 |

TABLE S2 Snow season candidate models for the probability of encountering a wolf (Prob. encounter) for woodland caribou in northeast British Columbia evaluated using Akaike’s Information Criteria for small sample sizes (AICc). Interactions indicated by *. Conifer swamp = conifswamp, hardswamp = hardwood swamp, nutrient-poor fen = poorfen, nutrient-rich fen = richfen, treed bog = bog, upland decidious = updecid, upland conifer = upconif, proportion of hardwood swamp within 100 m = hardswamp100, proportion of treed bog within 100 m = bog100, proportion of nutrient-poor fen within 100 m = poorfen100, nutrient-rich fen within 100 m = richfen100, terrain roughness (standard deviation of slopes) within 100 m = tr100, vegetation class diversity within 100 m = vcd100, density (m/km^2^) of vegetation class edges within 100 m = edge100, density (m/km^2^) of roads within 100 m = road100, distance (m) to road = distroad, density (m/km^2^) of seismic lines within 100 m = seismic100, distance (m) to seismic line = distseismic, distance (m) to vegetation class edge = distedge, distance (m) to water = distwater, number of parameters = k

| Prob. encounter models - snow | k | Log- likelihood | AICc | Delta AICc |
| --- | --- | --- | --- | --- |
| Prob. encounter=hardswamp100+bog100+elevation+distseismic+distroad | 6 | -417.2 | 846.5 | 0.0 |
| Prob. encounter=hardswamp100+bog100+distwater+elevation+tr100+seismic100+distseismic | 8 | -419.0 | 854.0 | 7.6 |
| Prob. encounter=hardswamp100+bog100+distwater+road100+distroad+seismic100+distseismic | 8 | -419.5 | 855.0 | 8.5 |
| Prob. encounter=hardswamp100+bog100+distroad+road100+distseismic+seismic100 | 7 | -421.0 | 856.0 | 9.5 |
| Prob. encounter=hardswamp100+bog100+elevation+seismic100+road100 | 6 | -422.8 | 857.5 | 11.0 |
| Prob. encounter=hardswamp100+bog100+distseismic+seismic100 | 5 | -424.2 | 858.5 | 12.0 |
| Prob. encounter=road100+distroad+distseismic+seismic100 | 5 | -424.8 | 859.7 | 13.2 |
| Prob. encounter=hardswamp100+bog100+distwater | 4 | -426.7 | 861.4 | 15.0 |
| Prob. encounter=hardswamp100+bog100+distwater+elevation+tr100+slope | 7 | -424.2 | 862.4 | 15.9 |
| Prob. encounter=hardswamp100+bog100 | 3 | -428.5 | 862.9 | 16.5 |
| Prob. encounter=(poorfen100*seismic100)+(richfen100*seismic100)+(bog100*seismic100) | 8 | -423.5 | 863.0 | 16.6 |
| Prob. encounter=slope+tr100+elevation | 4 | -427.9 | 863.9 | 17.4 |
| Prob. encounter=hardswamp100+bog100+vcd100 | 4 | -428.3 | 864.5 | 18.0 |
| Prob. encounter=hardswamp100+bog100+distwater+distedge+vcd100 | 6 | -426.5 | 865.0 | 18.5 |
| Prob. encounter=hardswamp100+bog100+distedge+edge100 | 5 | -427.5 | 865.0 | 18.5 |
| Prob. encounter=poorfen100+richfen100+bog100 | 4 | -429.6 | 867.2 | 20.7 |
| Prob. encounter=conifswamp+hardswamp+poorfen+richfen+bog+updecid+upconif | 8 | -428.7 | 873.4 | 26.9 |

TABLE S3. Snow-free season candidate models for the probability of being killed given an encounter (Prob. being killed) for woodland caribou in northeast British Columbia evaluated using Akaike’s Information Criteria for small sample sizes (AICc). Proportion of conifer and hardwood swamps within 100 m = swamp100, proportion of treed bog within 100 m = bog100, proportion of nutrient-poor and nutrient-rich fens within 100 m = fen100, terrain roughness (standard deviation of slopes) within 100 m = tr100, vegetation class diversity within 100 m = vcd100, density (m/km^2^) of vegetation class edges within 100 m = edge100, density (m/km^2^) of roads within 100 m = road100, density (m/km^2^) of seismic lines within 100 m = seismic100, number of parameters = k

| Prob. being killed models – snow-free | k | Log- likelihood | AICc | Delta AICc |
| --- | --- | --- | --- | --- |
| Prob. being killed=swamp100+edge100+tr100 | 4 | -37.5 | 83.5 | 0.0 |
| Prob. being killed=swamp100+tr100+vcd100 | 4 | -37.8 | 84.1 | 0.6 |
| Prob. being killed=swamp100+seismic100+edge100+tr100 | 5 | -36.9 | 84.7 | 1.2 |
| Prob. being killed=swamp100+seismic100+tr100+vcd100 | 5 | -37.4 | 85.6 | 2.1 |
| Prob. being killed=road100+seismic100+edge100+tr100 | 5 | -37.4 | 85.6 | 2.1 |
| Prob. being killed=swamp100+road100+seismic100 | 4 | -38.8 | 86.2 | 2.7 |
| Prob. being killed=road100+seismic100+tr100+vcd100 | 5 | -37.7 | 86.3 | 2.8 |
| Prob. being killed=swamp100+bog100+fen100+edge100+tr100 | 6 | -36.7 | 86.7 | 3.2 |
| Prob. being killed=swamp100+bog100+fen100+tr100+vcd100 | 6 | -36.8 | 86.8 | 3.3 |
| Prob. being killed=swamp100 | 2 | -41.9 | 88.0 | 4.5 |
| Prob. being killed=swamp100+bog100+fen100+road100+seismic100 | 6 | -37.5 | 88.2 | 4.7 |
| Prob. being killed=swamp100+bog100+fen100+seismic100+edge100+tr100 | 7 | -36.4 | 88.4 | 4.9 |
| Prob. being killed=swamp100+bog100+fen100+seismic100+tr100+vcd100 | 7 | -36.6 | 88.8 | 5.3 |
| Prob. being killed=swamp100+bog100+fen100 | 4 | -40.2 | 89.0 | 5.5 |
| Prob. being killed=road100+seismic100 | 3 | -41.3 | 89.0 | 5.5 |

TABLE S4. Snow-free season candidate models for the probability of being killed given an encounter (Prob. being killed) for woodland caribou in northeast British Columbia evaluated using Akaike’s Information Criteria for small sample sizes (AICc). Proportion of conifer and hardwood swamps within 500 m = swamp500, proportion of treed bog within 500 m = bog500, proportion of nutrient-poor and nutrient-rich fens within 500 m = fen500, terrain roughness (standard deviation of slopes) within 500 m = tr500, vegetation class diversity within 500 m = vcd500, density (m/km^2^) of vegetation class edges within 500 m = edge500, density (m/km^2^) of roads within 500 m = road500, density (m/km^2^) of seismic lines within 500 m = seismic500, number of parameters = k

| Prob. being killed models – snow-free | k | Log-likelihood | AICc | Delta AICc |
| --- | --- | --- | --- | --- |
| Prob. being killed=swamp500+edge500+tr500 | 4 | -35.2 | 78.9 | 0.0 |
| Prob. being killed=swamp500+tr500+vcd500 | 4 | -35.9 | 80.4 | 1.5 |
| Prob. being killed=swamp500+seismic500+edge500+tr500 | 5 | -35.2 | 81.2 | 2.3 |
| Prob. being killed=swamp500+seismic500+tr500+vcd500 | 5 | -35.9 | 82.6 | 3.7 |
| Prob. being killed=road500+seismic500+edge500+tr500 | 5 | -35.9 | 82.6 | 3.7 |
| Prob. being killed=swamp500+road500+seismic500 | 4 | -40.7 | 90.0 | 11.1 |
| Prob. being killed=road500+seismic500+tr500+vcd500 | 5 | -35.6 | 82.1 | 3.2 |
| Prob. being killed=swamp500+bog500+fen500+edge500+tr500 | 6 | -34.9 | 83.0 | 4.1 |
| Prob. being killed=swamp500+bog500+fen500+tr500+vcd500 | 6 | -35.8 | 84.7 | 5.8 |
| Prob. being killed=swamp500 | 2 | -41.9 | 87.9 | 9.0 |
| Prob. being killed=swamp500+bog500+fen500+road500+seismic500 | 6 | -36.6 | 86.5 | 7.6 |
| Prob. being killed=swamp500+bog500+fen500+seismic500+edge500+tr500 | 7 | -34.9 | 85.3 | 6.4 |
| Prob. being killed=swamp500+bog500+fen500+seismic500+tr500+vcd500 | 7 | -35.7 | 87.1 | 8.2 |
| Prob. being killed=swamp500+bog500+fen500 | 4 | -39.0 | 86.5 | 7.6 |
| Prob. being killed=road500+seismic500 | 3 | -43.0 | 92.2 | 13.3 |

TABLE S5. Snow season candidate models for the probability of being killed given an encounter (Prob. being killed) for woodland caribou in northeast British Columbia evaluated using Akaike’s Information Criteria for small sample sizes (AICc). Proportion of conifer and hardwood swamps within 100 m = swamp100, proportion of treed bog within 100 m = bog100, proportion of nutrient-poor and nutrient-rich fens within 100 m = fen100, terrain roughness (standard deviation of slopes) within 100 m = tr100, vegetation class diversity within 100 m = vcd100, density (m/km^2^) of vegetation class edges within 100 m = edge100, density (m/km^2^) of roads within 100 m = road100, density (m/km^2^) of seismic lines within 100 m = seismic100, number of parameters = k

| Prob. being killed models - snow | k | Log-likelihood | AICc | Delta AICc |
| --- | --- | --- | --- | --- |
| Prob. being killed=swamp100+bog100+fen100 | 4 | -52.2 | 113.0 | 0.0 |
| Prob. being killed=swamp100 | 2 | -55.0 | 114.3 | 1.3 |
| Prob. being killed=swamp100+bog100+fen100+tr100+vcd100 | 6 | -50.8 | 114.8 | 1.8 |
| Prob. being killed=swamp100+bog100+fen100+edge100+tr100 | 6 | -50.9 | 115.0 | 2.0 |
| Prob. being killed=swamp100+road100+seismic100 | 4 | -53.6 | 115.9 | 2.9 |
| Prob. being killed=swamp100+bog100+fen100+road100+seismic100 | 6 | -51.4 | 116.0 | 3.0 |
| Prob. being killed=swamp100+bog100+fen100+seismic100+tr100+vcd100 | 7 | -50.3 | 116.1 | 3.1 |
| Prob. being killed=swamp100+bog100+fen100+seismic100+edge100+tr100 | 7 | -50.3 | 116.2 | 3.2 |
| Prob. being killed=swamp100+tr100+vcd100 | 4 | -54.4 | 117.4 | 4.4 |
| Prob. being killed=swamp100+seismic100+tr100+vcd100 | 5 | -53.4 | 117.7 | 4.7 |
| Prob. being killed=swamp100+seismic100+edge100+tr100 | 5 | -53.5 | 117.9 | 4.9 |
| Prob. being killed=swamp100+edge100+tr100 | 4 | -54.7 | 118.0 | 5.0 |
| Prob. being killed=road100+seismic100 | 3 | -58.5 | 123.4 | 10.4 |
| Prob. being killed=road100+seismic100+tr100+vcd100 | 5 | -57.6 | 126.1 | 13.1 |
| Prob. being killed=road100+seismic100+edge100+tr100 | 5 | -58.1 | 127.1 | 14.1 |

TABLE S6. Snow season candidate models for the probability of being killed given an encounter (Prob. being killed) for woodland caribou in northeast British Columbia evaluated using Akaike’s Information Criteria for small sample sizes (AICc). Proportion of conifer and hardwood swamps within 500 m = swamp500, proportion of treed bog within 500 m = bog500, proportion of nutrient-poor and nutrient-rich fens within 500 m = fen500, terrain roughness (standard deviation of slopes) within 500 m = tr500, vegetation class diversity within 500 m = vcd500, density (m/km^2^) of vegetation class edges within 500 m = edge500, density (m/km^2^) of roads within 500 m = road500, density (m/km^2^) of seismic lines within 500 m = seismic500, number of parameters = k

| Prob. being killed models – snow | k | Log-likelihood | AICc | Delta AICc |
| --- | --- | --- | --- | --- |
| Prob. being killed=swamp500+bog500+fen500 | 4 | -54.8 | 118.0 | 0.0 |
| Prob. being killed=swamp500 | 2 | -58.3 | 120.7 | 2.7 |
| Prob. being killed=swamp500+bog500+fen500+tr500+vcd500 | 6 | -54.1 | 121.1 | 3.1 |
| Prob. being killed=swamp500+bog500+fen500+edge500+tr500 | 6 | -53.9 | 120.8 | 2.9 |
| Prob. being killed=swamp500+road500+seismic500 | 4 | -55.9 | 120.2 | 2.3 |
| Prob. being killed=swamp500+bog500+fen500+road500+seismic500 | 6 | -52.8 | 118.7 | 0.7 |
| Prob. being killed=swamp500+bog500+fen500+seismic500+tr500+vcd500 | 7 | -52.5 | 120.4 | 2.4 |
| Prob. being killed=swamp500+bog500+fen500+seismic500+edge500+tr500 | 7 | -52.4 | 120.2 | 2.2 |
| Prob. being killed=swamp500+tr500+vcd500 | 4 | -57.6 | 123.7 | 5.7 |
| Prob. being killed=swamp500+seismic500+tr500+vcd500 | 5 | -55.5 | 121.8 | 3.8 |
| Prob. being killed=swamp500+seismic500+edge500+tr500 | 5 | -56.0 | 122.7 | 4.7 |
| Prob. being killed=swamp500+edge500+tr500 | 4 | -58.2 | 124.9 | 7.0 |
| Prob. being killed=road500+seismic500 | 3 | -57.9 | 122.2 | 4.2 |
| Prob. being killed=road500+seismic500+tr500+vcd500 | 5 | -56.2 | 123.0 | 5.1 |
| Prob. being killed=road500+seismic500+edge500+tr500 | 5 | -57.5 | 125.6 | 7.7 |

TABLE S7. Comparing vegetation-class base models and vegetation-class models with risk covariates (predicted probability of encountering a wolf and predicted probability of being killed given an encounter) for boreal caribou in northeast British Columbia during calving, late summer, early winter, and late winter using Akaike’s Information Criteria for small sample sizes (AICc). Vegetation-classes included conifer swamp, hardwood swamp, poor fen, rich fen, treed bog, upland conifer, and upland deciduous. Number of parameters = k

| Season | Model | k | Log-likelihood | AICc | Delta AICc |
| --- | --- | --- | --- | --- | --- |
| Calving | Vegetation-class + risk covariates | 11 | -40970.3 | 81962.6 | 0.0 |
|  | Vegetation-class base model | 9 | -41010.2 | 82038.4 | 75.8 |
|  |  |  |  |  |  |
| Late summer | Vegetation-class + risk covariates | 11 | -65218.2 | 130458.4 | 0.0 |
|  | Vegetation-class base model | 9 | -65386.8 | 130791.6 | 333.2 |
|  |  |  |  |  |  |
| Early winter | Vegetation-class + risk covariates | 11 | -52925.4 | 105872.9 | 0.0 |
|  | Vegetation-class base model | 9 | -53672.3 | 107362.6 | 1489.7 |
|  |  |  |  |  |  |
| Late winter | Vegetation-class + risk covariates | 11 | -66513.5 | 133049.1 | 0.0 |
|  | Vegetation-class base model | 9 | -67314.1 | 134646.2 | 1597.1 |

TABLE S8. Coefficients (β), standard errors (SE), test-statistics (T-value), and p-values from general linear regression models relating individual coefficients for the predicted probability of encountering a wolf (from resource selection functions) during calving, late summer, early winter, and late winter to age and reproductive status (calf or no calf) for boreal caribou in northeast British Columbia. When only one covariate was significant, we excluded the non-significant covariate and ran univariate models including the remaining significant covariate.

|  |  | Calving | | | |
| --- | --- | --- | --- | --- | --- |
| Model | Covariate | β | SE | T-value | P-value |
| Multivariate | Intercept | 0.124 | 0.114 | 1.086 | 0.280 |
|  | Age | -0.034 | 0.018 | -1.912 | 0.058 |
|  | No calf | 0.049 | 0.140 | 0.353 | 0.725 |
|  |  |  |  |  |  |
| Univariate | Intercept | 0.136 | 0.108 | 1.261 | 0.209 |
|  | Age | -0.035 | 0.017 | -2.071 | 0.040 |
|  |  |  |  |  |  |
|  |  | Late summer | | | |
| Model | Covariate | β | SE | T-value | P-value |
| Multivariate | Intercept | 0.458 | 0.149 | 3.080 | 0.003 |
|  | Age | -0.071 | 0.024 | -3.028 | 0.003 |
|  | Calf | -0.243 | 0.134 | -1.815 | 0.072 |
|  |  |  |  |  |  |
|  |  | Early winter | | | |
| Model | Covariate | β | SE | T-value | P-value |
| Multivariate | Intercept | 0.150 | 0.081 | 1.845 | 0.067 |
|  | Age | -0.023 | 0.013 | -1.826 | 0.070 |
|  | Calf | -0.082 | 0.072 | -1.143 | 0.255 |
|  |  |  |  |  |  |
| Univariate | Intercept | 0.143 | 0.081 | 1.762 | 0.080 |
|  | Age | -0.024 | 0.013 | -1.881 | 0.062 |
|  |  |  |  |  |  |
|  |  | Late winter | | | |
| Model | Covariate | β | SE | T-value | P-value |
| Multivariate | Intercept | 0.018 | 0.075 | 0.243 | 0.808 |
|  | Age | -0.007 | 0.012 | -0.608 | 0.544 |
|  | Calf | -0.063 | 0.078 | -0.808 | 0.420 |

TABLE S9. Coefficients (β), standard errors (SE), test-statistics (T-value), and p-values from general linear regression models relating individual coefficients for the predicted probability of being killed given a wolf encounter (from resource selection functions) during calving, late summer, early winter, and late winter to age and reproductive status (calf or no calf) for boreal caribou in northeast British Columbia. When only one covariate was significant, we excluded the non-significant covariate and ran univariate models including the remaining significant covariate.

|  |  | Calving | | | |
| --- | --- | --- | --- | --- | --- |
| Model | Covariate | β | SE | T-value | P-value |
| Multivariate | Intercept | -1.779 | 0.388 | -4.590 | <0.001 |
|  | Age | 0.055 | 0.060 | 0.922 | 0.358 |
|  | No calf | -0.232 | 0.476 | -0.487 | 0.627 |
|  |  |  |  |  |  |
|  |  | Late summer | | | |
| Model | Covariate | β | SE | T-value | P-value |
| Multivariate | Intercept | -2.217 | 0.327 | -6.784 | <0.001 |
|  | Age | 0.090 | 0.052 | 1.734 | 0.085 |
|  | Calf | 0.646 | 0.295 | 2.189 | 0.030 |
|  |  |  |  |  |  |
|  |  | Early winter | | | |
| Model | Covariate | β | SE | T-value | P-value |
| Multivariate | Intercept | -1.889 | 0.273 | -6.919 | <0.001 |
|  | Age | 0.002 | 0.042 | 0.048 | 0.962 |
|  | Calf | -0.159 | 0.242 | -0.658 | 0.512 |
|  |  |  |  |  |  |
|  |  | Late winter | | | |
| Model | Covariate | β | SE | T-value | P-value |
| Multivariate | Intercept | -1.717 | 0.312 | -5.502 | <0.001 |
|  | Age | 0.025 | 0.049 | 0.506 | 0.614 |
|  | Calf | 0.227 | 0.323 | 0.702 | 0.484 |

TABLE S10. Comparing vegetation-class base models and vegetation-class models with density (m/km^2^) and distance (m) covariates (densities of roads and seismic lines and distances to roads and seismic lines) for boreal caribou in northeast British Columbia during calving, late summer, early winter, and late winter using Akaike’s Information Criteria for small sample sizes (AICc). Vegetation-classes included conifer swamp, hardwood swamp, poor fen, rich fen, treed bog, upland conifer, and upland deciduous. Number of parameters = k

| Season | Model | k | Log-likelihood | AICc | Delta AICc |
| --- | --- | --- | --- | --- | --- |
| Calving | Vegetation-class + distance covariates | 11 | -40804.8 | 81631.5 | 0.0 |
|  | Vegetation-class + density covariates | 11 | -40868.0 | 81758.0 | 126.5 |
|  | Vegetation-class base model | 9 | -41010.2 | 82038.4 | 406.9 |
|  |  |  |  |  |  |
| Late summer | Vegetation-class + distance covariates | 11 | -65295.5 | 130613.0 | 0.0 |
|  | Vegetation-class + density covariates | 11 | -65302.5 | 130627.0 | 14.0 |
|  | Vegetation-class base model | 9 | -65386.8 | 130791.6 | 178.5 |
|  |  |  |  |  |  |
| Early winter | Vegetation-class + density covariates | 11 | -53577.7 | 107177.4 | 0.0 |
|  | Vegetation-class + distance covariates | 11 | -53627.8 | 107277.6 | 500.3 |
|  | Vegetation-class base model | 9 | -53672.3 | 107362.6 | 185.2 |
|  |  |  |  |  |  |
| Late winter | Vegetation-class + density covariates | 11 | -67124.8 | 134271.6 | 0.0 |
|  | Vegetation-class + distance covariates | 11 | -67238.1 | 134498.2 | 226.5 |
|  | Vegetation-class base model | 9 | -67314.1 | 134646.2 | 374.6 |

TABLE S11. The coefficients (β) and standard errors (SE) of fixed effects and the variance (σ) of random effects in resource selection functions including the densities (m/km^2^) of roads and seismic lines (Seismic) during calving, late summer, early winter, and late winter seasons for boreal caribou in northeast British Columbia. Nutrient-poor fen = poor fen, nutrient-rich fen = rich fen

|  | Calving | | Late summer | |
| --- | --- | --- | --- | --- |
| Fixed effects | β | SE | β | SE |
| Conifer swamp | 0.158 | 0.038 | 0.164 | 0.029 |
| Hardwood swamp | 0.000 | 0.039 | -0.316 | 0.032 |
| Poor fen | 0.730 | 0.024 | 0.593 | 0.017 |
| Rich fen | 0.465 | 0.034 | 0.190 | 0.028 |
| Treed bog | 0.747 | 0.024 | 0.715 | 0.017 |
| Upland conifer | -0.548 | 0.059 | -0.330 | 0.041 |
| Upland deciduous | -1.353 | 0.096 | -1.400 | 0.064 |
|  |  |  |  |  |
| Random effects | σ |  | σ |  |
| Road density | 8.961 |  | 4.033 |  |
| Seismic density | 3.132 |  | 0.670 |  |
| Individual | 0.008 |  | 0.008 |  |
|  |  |  |  |  |
|  | Early winter | | Late winter | |
| Fixed effects | β | SE | β | SE |
| Conifer swamp | -0.145 | 0.037 | -0.003 | 0.029 |
| Hardwood swamp | -0.224 | 0.035 | -0.406 | 0.035 |
| Poor fen | 0.344 | 0.021 | 0.552 | 0.018 |
| Rich fen | 0.505 | 0.028 | 0.036 | 0.029 |
| Treed bog | 0.960 | 0.019 | 0.919 | 0.017 |
| Upland conifer | -0.340 | 0.048 | -0.131 | 0.038 |
| Upland deciduous | -1.620 | 0.077 | -1.263 | 0.062 |
|  |  |  |  |  |
| Random effects | σ |  | σ |  |
| Road density | 2.562 |  | 7.022 |  |
| Seismic density | 0.668 |  | 1.051 |  |
| Individual | 0.015 |  | 0.025 |  |

Table S12. The coefficients (β) and standard errors (SE) of fixed effects and the variance (σ) of random effects in resource selection functions including the distances (Dist.) to road and seismic lines (Seismic) during calving, late summer, early winter, and late winter seasons for boreal caribou in northeast British Columbia. Nutrient-poor fen = poor fen, nutrient-rich fen = rich fen

|  | Calving | | Late summer | |
| --- | --- | --- | --- | --- |
| Fixed effects | β | SE | β | SE |
| Conifer swamp | 0.148 | 0.038 | 0.163 | 0.030 |
| Hardwood swamp | 0.019 | 0.039 | -0.302 | 0.032 |
| Poor fen | 0.747 | 0.024 | 0.599 | 0.017 |
| Rich fen | 0.466 | 0.035 | 0.189 | 0.028 |
| Treed bog | 0.779 | 0.024 | 0.738 | 0.017 |
| Upland conifer | -0.587 | 0.060 | -0.381 | 0.042 |
| Upland deciduous | -1.412 | 0.097 | -1.415 | 0.064 |
|  |  |  |  |  |
| Random effects | σ |  | σ |  |
| Dist. to road | 17.606 |  | 3.621 |  |
| Dist. to seismic | 5.720 |  | 0.089 |  |
| Individual | 0.097 |  | 0.013 |  |
|  |  |  |  |  |
|  | Early winter | | Late winter | |
| Fixed effects | β | SE | β | SE |
| Conifer swamp | -0.163 | 0.037 | -0.002 | 0.029 |
| Hardwood swamp | -0.229 | 0.035 | -0.399 | 0.035 |
| Poor fen | 0.342 | 0.021 | 0.558 | 0.018 |
| Rich fen | 0.509 | 0.028 | 0.035 | 0.029 |
| Treed bog | 0.967 | 0.019 | 0.937 | 0.017 |
| Upland conifer | -0.361 | 0.048 | -0.136 | 0.038 |
| Upland deciduous | -1.627 | 0.077 | -1.282 | 0.062 |
|  |  |  |  |  |
| Random effects | σ |  | σ |  |
| Dist. to road | 1.690 |  | 3.333 |  |
| Dist. to seismic | 1.000 |  | 0.034 |  |
| Individual | 0.029 |  | 0.028 |  |

TABLE S13. Coefficients (β), standard errors (SE), test-statistics (T-value), and p-values from general linear regression models relating individual coefficients for the density of roads (from resource selection functions) during calving, late summer, early winter, and late winter to age and reproductive status (calf or no calf) for boreal caribou in northeast British Columbia. When only one covariate was significant, we excluded the non-significant covariate and ran univariate models including the remaining significant covariate.

|  |  | Calving | | | |
| --- | --- | --- | --- | --- | --- |
| Model | Covariate | β | SE | T-value | P-value |
| Multivariate | Intercept | -0.188 | 0.499 | -0.377 | 0.707 |
|  | Age | -0.222 | 0.077 | -2.891 | 0.004 |
|  | No calf | 1.431 | 0.614 | 2.332 | 0.021 |
|  |  |  |  |  |  |
|  |  | Late summer | | | |
| Model | Covariate | β | SE | T-value | P-value |
| Multivariate | Intercept | 0.366 | 0.381 | 0.960 | 0.339 |
|  | Age | -0.174 | 0.060 | -2.872 | 0.005 |
|  | Calf | -0.764 | 0.344 | -2.219 | 0.028 |
|  |  |  |  |  |  |
|  |  | Early winter | | | |
| Model | Covariate | β | SE | T-value | P-value |
| Multivariate | Intercept | 0.984 | 0.301 | 3.274 | 0.001 |
|  | Age | -0.160 | 0.047 | -3.417 | 0.001 |
|  | Calf | 0.145 | 0.266 | 0.544 | 0.587 |
|  |  |  |  |  |  |
| Univariate | Intercept | 0.996 | 0.299 | 3.333 | 0.001 |
|  | Age | -0.158 | 0.047 | -3.403 | 0.001 |
|  |  |  |  |  |  |
|  |  | Late winter | | | |
| Model | Covariate | β | SE | T-value | P-value |
| Multivariate | Intercept | -0.705 | 0.523 | -1.348 | 0.180 |
|  | Age | -0.058 | 0.081 | -0.711 | 0.478 |
|  | Calf | 0.369 | 0.541 | 0.681 | 0.497 |

TABLE S14. Coefficients (β), standard errors (SE), test-statistics (T-value), and p-values from general linear regression models relating individual coefficients for the distance to roads (from resource selection functions) during calving, late summer, early winter, and late winter to age and reproductive status (calf or no calf) for boreal caribou in northeast British Columbia. When only one covariate was significant, we excluded the non-significant covariate and ran univariate models including the remaining significant covariate.

|  |  | Calving | | | |
| --- | --- | --- | --- | --- | --- |
| Model | Covariate | β | SE | T-value | P-value |
| Multivariate | Intercept | -0.1219 | 1.0479 | -0.116 | 0.9076 |
|  | Age | 0.2309 | 0.1609 | 1.435 | 0.1535 |
|  | No calf | -2.2221 | 1.2877 | -1.726 | 0.0866 |
|  |  |  |  |  |  |
| Univariate | Intercept | 1.313 | 0.314 | 4.182 | <0.001 |
|  | No calf | -2.683 | 1.252 | -2.143 | 0.034 |
|  |  |  |  |  |  |
|  |  | Late summer | | | |
| Model | Covariate | β | SE | T-value | P-value |
| Multivariate | Intercept | -0.369 | 0.445 | -0.829 | 0.409 |
|  | Age | 0.137 | 0.071 | 1.949 | 0.053 |
|  | Calf | 0.394 | 0.402 | 0.980 | 0.329 |
|  |  |  |  |  |  |
| Univariate | Intercept | -0.331 | 0.443 | -0.746 | 0.457 |
|  | Age | 0.140 | 0.071 | 1.979 | 0.050 |
|  |  |  |  |  |  |
|  |  | Early winter | | | |
| Model | Covariate | β | SE | T-value | P-value |
| Multivariate | Intercept | -0.009 | 0.251 | -0.036 | 0.971 |
|  | Age | 0.038 | 0.039 | 0.978 | 0.330 |
|  | Calf | -0.317 | 0.222 | -1.428 | 0.155 |
|  |  |  |  |  |  |
|  |  | Late winter | | | |
| Model | Covariate | β | SE | T-value | P-value |
| Multivariate | Intercept | 0.399 | 0.416 | 0.960 | 0.339 |
|  | Age | -0.004 | 0.065 | -0.061 | 0.952 |
|  | Calf | -0.697 | 0.430 | -1.619 | 0.107 |

TABLE S15. Coefficients (β), standard errors (SE), test-statistics (T-value), and p-values from general linear regression models relating individual coefficients for the density of seismic lines (from resource selection functions) during calving, late summer, early winter, and late winter to age and reproductive status (calf or no calf) for boreal caribou in northeast British Columbia. When only one covariate was significant, we excluded the non-significant covariate and ran univariate models including the remaining significant covariate.

|  |  | Calving | | | |
| --- | --- | --- | --- | --- | --- |
| Model | Covariate | β | SE | T-value | P-value |
| Multivariate | Intercept | -0.160 | 0.281 | -0.570 | 0.569 |
|  | Age | -0.054 | 0.043 | -1.254 | 0.212 |
|  | No calf | 0.618 | 0.345 | 1.791 | 0.075 |
|  |  |  |  |  |  |
| Univariate | Intercept | -0.496 | 0.084 | -5.907 | <0.001 |
|  | No calf | 0.726 | 0.335 | 2.168 | 0.032 |
|  |  |  |  |  |  |
|  |  | Late summer | | | |
| Model | Covariate | β | SE | T-value | P-value |
| Multivariate | Intercept | 0.079 | 0.125 | 0.634 | 0.527 |
|  | Age | -0.026 | 0.020 | -1.311 | 0.192 |
|  | Calf | -0.125 | 0.113 | -1.107 | 0.270 |
|  |  |  |  |  |  |
|  |  | Early winter | | | |
| Model | Covariate | β | SE | T-value | P-value |
| Multivariate | Intercept | 0.13934 | 0.11285 | 1.235 | 0.219 |
|  | Age | -0.0173 | 0.01753 | -0.988 | 0.325 |
|  | Calf | -0.1067 | 0.09985 | -1.068 | 0.287 |
|  |  |  |  |  |  |
|  |  | Late winter | | | |
| Model | Covariate | β | SE | T-value | P-value |
| Multivariate | Intercept | -0.295 | 0.149 | -1.978 | 0.050 |
|  | Age | 0.015 | 0.023 | 0.627 | 0.532 |
|  | Calf | 0.124 | 0.155 | 0.801 | 0.424 |

TABLE S16. Coefficients (β), standard errors (SE), test-statistics (T-value), and p-values from general linear regression models relating individual coefficients for the distance to seismic lines (from resource selection functions) during calving, late summer, early winter, and late winter to age and reproductive status (calf or no calf) for boreal caribou in northeast British Columbia.

|  |  | Calving | | | |
| --- | --- | --- | --- | --- | --- |
| Model | Covariate | β | SE | T-value | P-value |
| Multivariate | Intercept | -0.5544 | 0.53176 | -1.043 | 0.299 |
|  | Age | 0.07928 | 0.08164 | 0.971 | 0.333 |
|  | No calf | -1.0295 | 0.65346 | -1.575 | 0.117 |
|  |  |  |  |  |  |
|  |  | Late summer | | | |
| Model | Covariate | β | SE | T-value | P-value |
| Multivariate | Intercept | 0.079 | 0.125 | 0.634 | 0.527 |
|  | Age | -0.026 | 0.020 | -1.311 | 0.192 |
|  | Calf | -0.125 | 0.113 | -1.107 | 0.270 |
|  |  |  |  |  |  |
|  |  | Early winter | | | |
| Model | Covariate | β | SE | T-value | P-value |
| Multivariate | Intercept | -0.216 | 0.171 | -1.266 | 0.208 |
|  | Age | 0.018 | 0.026 | 0.691 | 0.491 |
|  | Calf | 0.083 | 0.151 | 0.548 | 0.584 |
|  |  |  |  |  |  |
|  |  | Late winter | | | |
| Model | Covariate | β | SE | T-value | P-value |
| Multivariate | Intercept | -0.004 | 0.011 | -0.383 | 0.702 |
|  | Age | 0.001 | 0.002 | 0.623 | 0.534 |
|  | Calf | 0.013 | 0.011 | 1.181 | 0.240 |
